# Diagnostic Accuracy of Urinary Biomarkers in Periodontitis: A Systematic Review and Meta-Analysis

**DOI:** 10.1155/2024/9769772

**Published:** 2024-07-29

**Authors:** Adriano Fratini, Rossana Izzetti, Nicola Riccetti, Stefano Gennai, Filippo Graziani, Enrico Marchetti

**Affiliations:** ^1^ Department of Life, Health and Environmental Sciences University of L'Aquila, L'Aquila 67100, Italy; ^2^ Department of Surgical, Medical and Molecular Pathology and Critical Care Medicine University of Pisa, Pisa 56126, Italy; ^3^ Institute of Medical Biostatistics Epidemiology and Informatics University Medical Centre Johannes Gutenberg University Mainz, Mainz, Germany

## Abstract

**Background:**

Biomarkers can be measured in various biological samples. Urine is among the most useful biofluids for routine testing, and several experimental and clinical studies support its role as a tool for the diagnosis and prevention of various diseases. The present systematic review aimed to examine periodontitis-specific urine biomarkers that could have a diagnostic relevance and to provide a qualitative assessment of the current literature.

**Materials and Methods:**

Relevant studies identified from PubMed, Embase, Cochrane Library, and Scopus databases were examined to answer the following PECO question: “Could the concentration of specific metabolites in the urine be related to periodontal health and what is their diagnostic accuracy?”. Quality of included studies was rated using ROBINS-I tool. Meta-analysis was conducted on available quantitative data.

**Results:**

After the screening of 768 titles, five studies were included in qualitative synthesis. The studies included referred to the evaluation of 8-hydroxy-2′-deoxyguanosine (8-OHdG) and neopterin. Meta-analysis was conducted for neopterin concentration on data available in four studies involving 129 participants. Higher concentrations of neopterin were found in periodontitis-affected patients compared to controls and patients treated for periodontitis.

**Conclusions:**

The literature appears controversial in attributing a role to neopterin and 8-OHdG as periodontal biomarkers, highlighting the need for further clinical studies on this topic. While some studies report variations in 8-OHdG and neopterin levels in periodontally affected patients versus either controls or periodontally treated patients, the level of evidence appears still limited to draw firm conclusions (PROSPERO CRD42020222681).

## 1. Introduction

Periodontitis is a multifactorial, chronic, inflammatory disease which recognizes as a major etiologic factor the presence of a dysbiotic plaque biofilm [1]. In terms of frequency, periodontitis is considered the most common chronic inflammatory disease in humans and the sixth most prevalent condition in the world [2]. Severe periodontitis is estimated to affect approximately 7%–11% of adults worldwide, while mild periodontitis affects around 50% of adults [2, 3].

The diagnosis of periodontitis relies on the evaluation of biometric clinical parameters, including probing depth (PD), clinical attachment level (CAL), and bleeding on probing (BOP) [4]. However, the assessment of the levels of inflammation through clinical parameters may not provide a complete overview of the current disease activity or future risk of breakdown [5].

From this perspective, an increasing application of biomarkers has been promoted to screen and predict the early onset of periodontitis. Biomarkers are biologic substances serving as indicators of biological health, pathogenic processes, environmental exposure, and pharmacologic responses to a therapeutic intervention [6]. Biomarkers can be measured in various biological samples, such as blood, urine, saliva, hair, faeces, cerebrospinal fluid, and body tissues. Urine is among the most useful biofluids for routine testing, and several experimental and clinical studies support its role as tool for the diagnosis, prevention, and treatment of various diseases, such as cancer, kidney diseases, infectious diseases, autoimmune diseases, and cardiovascular diseases [7].

Interestingly, evidence from the literature supports the assessment of biological diagnostic markers as indicators of periodontal status. Gingival crevicular fluid (GCF) and saliva have been claimed as potential repositories for the molecular changes associated with the destruction of periodontal tissues [8]. GCF is considered relevant for the assessment of the status of periodontal tissues, being rich in antibodies against oral microorganisms present in the dental biofilm, inflammatory mediators and tissue breakdown products [9]. Salivary biomarkers encompass a wide variety of inflammation-related cytokines and immunoglobulins, which can be indicative of the presence of periodontitis [10]. Consistently with the growing body of evidence on the relationship between periodontitis and systemic inflammation, the assessment of blood C-reactive protein (CRP) fluctuations in course of periodontitis, or following its treatment, have shed a light on the relationship of biomarkers in course of chronic periodontal diseases [11]. Although the relationship between blood and salivary biomarkers and periodontitis has been extensively studied, urine testing has only recently been suggested as a possible method for assessing periodontal health [12].

The aim of the present study was to systematically review the evidence on urine biomarkers in course of periodontitis and to assess their diagnostic relevance through a qualitative assessment of the current scientific literature.

## 2. Materials and Methods

### 2.1. Study Protocol

This research was conducted in accordance with the Cochrane Handbook and reported according to the PRISMA guidelines (Table 1) [36, 37, 38]. The protocol was registered at the International Prospective Register of Systematic Reviews (PROSPERO), registration number: CRD42020222681.

The following focused question was phrased:

“Could the concentration of specific metabolites in the urine be related to periodontal health and what is their diagnostic accuracy?”

Articles to be included had to follow the following PECO:

  (P) Population. Adult systemically healthy patients.
  (E) Exposure. Patients or sites with a clinical or radiographic diagnosis of periodontitis.
  (C) Comparison. Subjects or sites treated for periodontitis, or periodontally healthy subjects.
  (O) Type of outcome measures. Changes in urinary metabolite concentration in periodontitis compared to treated groups or healthy subjects.

No time limitations were applied. Only articles in English were included.

### 2.2. Information Sources and Search

An electronic search was conducted by two independent reviewers (AF, LM) using PubMed, Embase, Cochrane Library, and Scopus for publications up to January 2022, using combinations of controlled terms (MeSH) and free text words. The search strategy was first designed for the MEDLINE database and was then modified for the other databases (Table 3). Additionally, a manual search of periodontology-related journals including the *Journal of Dental Research*, the*Journal of Clinical Periodontology*, the *Journal of Periodontal Research*, and the *Journal of Periodontology* was performed from 2010 to 2021. All references were exported and managed in the open-access platform Colandr (https://www.colandrapp.com) [39].

### 2.3. Eligibility criteria

The inclusion criteria for title and abstract analysis are the following:

1. Studies reporting on systemically healthy human subjects affected by periodontitis.
2. Collection of urine metabolomic biomarkers.
3. Study design: RCTs and clinical trials.

Exclusion criteria are as follows:

1. Studies reporting on periodontally affected adult patients with systemic diseases and/or pregnant or breastfeeding.
2. Study design: Case reports, literature reviews, editorials, animal studies, and in vitro experiments.

### 2.4. Study Selection

The titles and abstracts of potentially eligible studies were screened independently by two reviewers (AF and LM). Any disagreement was resolved by discussion with a third reviewer (EM). Cohen's K-score was calculated to assess the interreviewer reliability in the screening phase.

The reasons for exclusion of studies after full-text analysis were recorded and reported (Table 2). Relevant articles that met the inclusion criteria were analyzed in full-text.

### 2.5. Data Extraction and Management

The following data were collected for each study (AF, LM): authors, study design, location, sample size, characteristics of patients (mean age, gender), periodontal diagnosis, treatment (if any), follow-up, urinary biomarkers and concentration levels, mean clinical outcomes (PPD, CAL), methodology used for sampling, storage, processing and detection of the urinary markers, and main findings.

### 2.6. Risk of Bias in the Included Studies and Quality Assessment

The quality assessment and the risk of bias of the included studies were performed independently by two calibrated reviewers (LM, AF). The risk of bias for nonrandomized studies was performed using ROBINS-I tool [40, 41]. In cases of critical or serious judgment, the study was considered at high risk of bias.

### 2.7. Data Synthesis

The urinary concentrations of biomarkers considered relevance for periodontal patients were reported. The concentration of each biomarker in periodontal patients at diagnosis/baseline and after treatment was expressed as mean and standard deviation.

A random-effect model was employed to estimate the average urinary mean concentration of each biomarker at diagnosis/baseline. Then, a random-effect model was applied to assess mean differences in urinary concentration of each biomarker between periodontal patients at diagnosis versus healthy controls, as well as between periodontal patients at diagnosis versus at first follow-up.

In all the models, a restricted maximum-likelihood estimator (REML) was used to estimate tau [2]. Tau [2] and prediction intervals were reported to represent the overall level of heterogeneity, as well as *I*^2^ to describe it. A value of *I*^2^ between 25% and 50% was considered as low heterogeneity, between 50% and 75% as medium heterogeneity, and >75% as high heterogeneity. The results of the meta-analyses were graphically represented using forest plots.

A meta-regression of the average urinary mean concentration of each biomarker at diagnosis/baseline was also performed, with mean age and gender as potential predictors.

The numerical synthesis of the results as well as the figures were developed using R studio and the metaphor package [42].

## 3. Results

### 3.1. Study Selection

In total, 768 articles were retrieved through the electronic search. Manual search did not retrieve further studies. After the removal of duplicates, 597 titles and abstracts were screened for eligibility and 25 papers were analyzed in full-text. Of these, 19 articles were excluded (Table 2), and six papers were included in the review (Figure 6). Inter-reviewer agreement was *k* = 0.92 for abstract screening and *k* = 0.97 for full-text analysis.

### 3.2. Study Characteristics

All the identified studies were published between 1997 and 2022 [30, 31, 32, 33, 34, 35]. Two studies were conducted in Turkey [30, 32], the others in Spain [35], India [33], Austria [34], and Czech Republic [31]. The sample size ranged between 16 and 70 individuals. All the papers reported observational studies. Two studies had a cross-sectional design [33, 35], three were case-control studies [30, 31, 32], and one was a cohort study [34].

Periodontitis case definitions and periodontal parameters were heterogeneous across the studies. Only two studies presented the classification for periodontal diseases currently in use [31, 35]. Two articles enrolled patients with chronic periodontitis (CP) [33, 34], and two studies referred to patients with aggressive periodontitis (AgP) [33, 34]. Three studies included a control group of healthy patients (Table 4) [30, 31, 32].

### 3.3. Synthesis of the Results

The studies included provided data on 8-hydroxy-2′-deoxyguanosine (8-OHdG) [35] and neopterin concentration [30, 31, 32, 33, 34] in a total of 199 patients (70 evaluated for 8-OHdG levels, 129 for neopterin levels). Meta-analysis was conducted only for neopterin, for which data were available in five studies involving 129 participants.

Gender was employed as a potential predictor for the meta-regression. Female gender in the sample was a significant predictor of the estimation of the average mean urinary concentration of neopterin (*p*=0.03). Female gender accounted for almost 60% of heterogeneity in the model (*R*^2^ = 59.39). Due to the limited number of observations and the missing values in the included studies, no meta-regression was conducted using the average age of the patients as potential predictor.

Regarding other confounders, only one study [35] included smokers, who represented 34.3% of the sample. Three studies [31, 32, 33] excluded smokers from the sample. The remaining two studies [30, 34] did not give any information on smoking status. Two studies [30, 33] included only systemically healthy patients. The remaining studies [31, 32, 34, 35] did not provide information on systemic health status.

### 3.4. Average Mean Concentration of 8-OHdG

The concentration of 8-OHdG was investigated in one study [35]. No differences were observed in 8-OHdG urine levels between groups of patients affected by different stages of periodontitis (10.9 ± 3.05 *μ*g/g in Stage II; 9.17 ± 2.05 *μ*g/g in Stage III; and 9.84 ± 3.21 *μ*g/g in Stage IV).

### 3.5. Average Mean Concentration of Neopterin

An average mean urinary concentration of neopterin of 195.75 mmol neopterin/mol creatinine (95% CI [100.30 to 291.20]; *p*=0.03; *I*^2^ = 61%) was found in periodontal patients at diagnosis (Figure 2).

When comparing periodontal patients and controls, controls showed lower neopterin levels, although no statistically significant differences were reported (ns = 3; np = 95; WMD = −18.88 mmol neopterin/mol creatinine; 95% CI [−44.06; 6.30]; *p*=0.82; *I*^2^ = 31.75%) (Figure 3).

Following periodontal treatment, a statistically significant decrease in neopterin levels was recorded (ns = 3; np = 63; WMD = −33.42 mmol neopterin/mol creatinine; 95% CI [−205.08; 138.23]; *p* < 0.01; *I*^2^ = 98%) (Figure 4).

### 3.6. Risk of Bias Assessment

**Figure 1:**
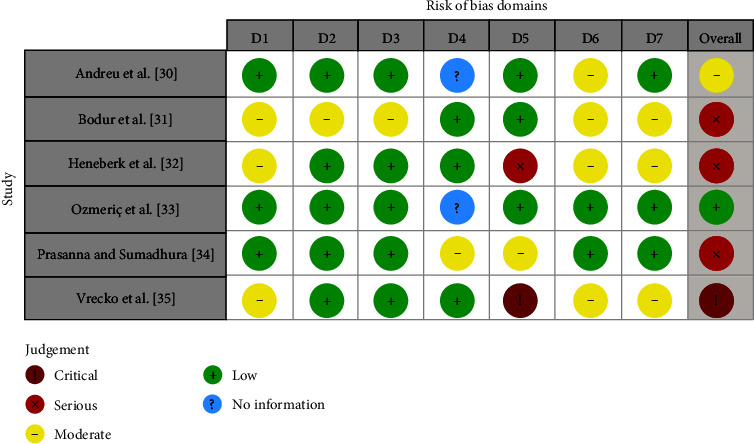
Risk of bias assessment for each of the included studies. Domains: D1, bias due to confounding; D2, bias due to selection of participants; D3, bias in classification of interventions; D4, bias due to deviations from intended interventions; D5, bias due to missing data; D6, bias in measurements of outcomes; D7, bias in selection of the reported result.

**Figure 2:**
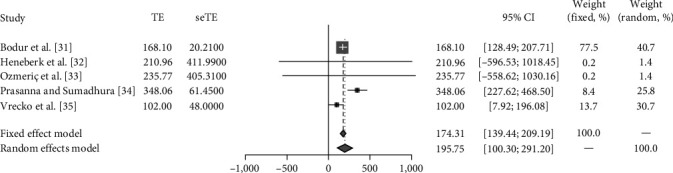
Meta-analysis of the four studies [31, 32, 33, 34, 35] reporting fluctuations in neopterin levels.

**Figure 3:**
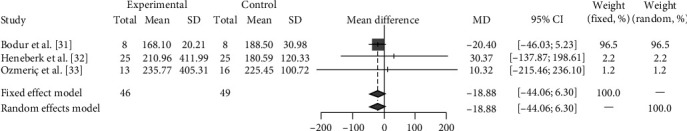
Meta-analysis of case-control studies [31, 32, 33].

**Figure 4:**
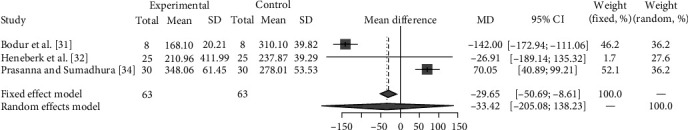
Meta-analysis of the studies [31, 32, 34] evaluating neopterin levels in periodontal patients pre- and post-treatment.

**Figure 5:**
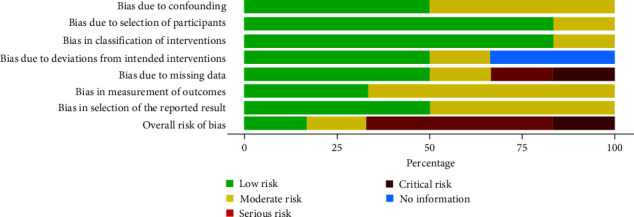
Risk of bias assessment with the ROBINS-I tool.

**Figure 6:**
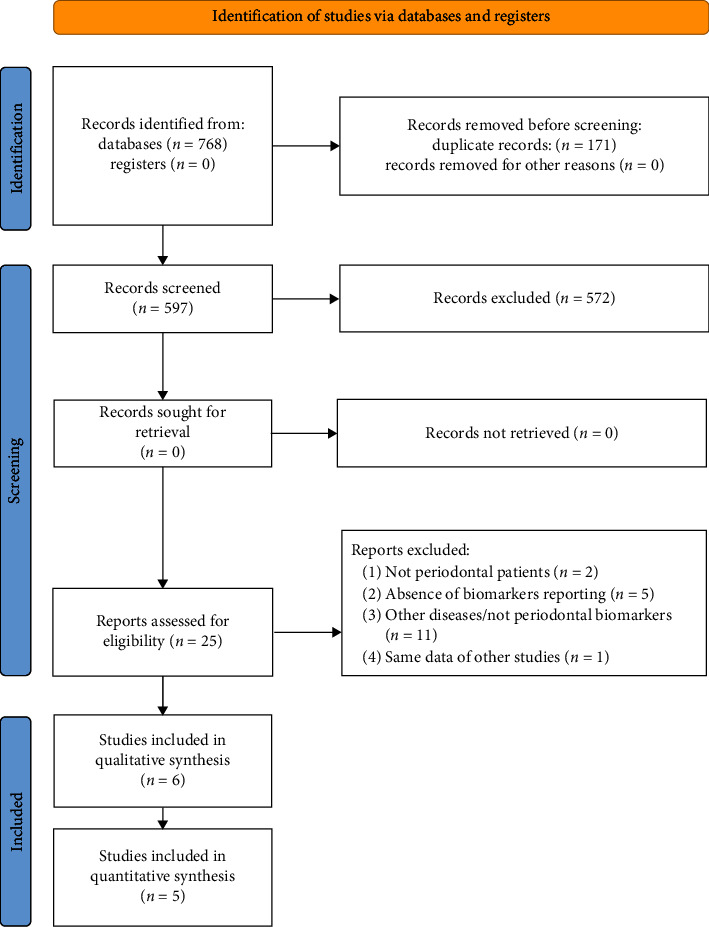
PRISMA flowchart. PRISMA 2020 flow diagram for new systematic reviews which included searches of databases and registers. From: MJ, McKenzie JE, Bossuyt PM, Boutron I, Hoffmann TC, Mulrow CD, et al. The PRISMA 2020 statement: an updated guideline for reporting systematic reviews. *BMJ* 2021;372:n71. doi: 10.1136/bmj.n71. For more information, visit at http://www.prisma-statement.org/.

The risk of bias assessment for the included studies was completed independently by two reviewers (AF, LM) as part of the data extraction process using ROBINS-I tool and is summarized in Figures 1 and 5. One paper [32] showed a low risk of bias, one paper [35] a moderate risk of bias, and three papers [30, 31, 33] a high risk of bias, while one paper [34] showed a critical risk of performance bias due to missing data.

## 4. Discussion

The present systematic review highlights an overall paucity of literature on the association between urine biomarkers and periodontal health status. The results suggest the presence of higher urine biomarkers concentrations in periodontally affected patients compared to treated patients or healthy controls. Neopterin may be a relevant biomarker of periodontal status, as its concentration is significantly lower both in healthy subjects and in patients receiving periodontal treatment. The evidence on 8-OHdG is extremely scarce, and no statistically significant differences were detected depending on the stage of periodontitis. It should be noted, though, that only one study investigated this biomarker and presented an overall limited study sample. Such results should be interpretated with caution due to the weak evidence provided by the literature available.

Periodontal biomarkers are a trending topic in the current literature. The increasing attention toward periodontal medicine has fostered the research on the systemic implications of periodontal diseases. Importantly, a relationship has been found between periodontitis and cardiovascular diseases [43], diabetes [44], adverse pregnancy outcomes [45], and in general with systemic inflammation [46]. Indeed, untreated periodontitis has been associated with elevated systemic inflammation, which could contribute to the development of systemic health complications [47]. Among the inflammation biomarkers studied in relationship to periodontitis, serum C-reactive protein (CRP) is the most commonly examined. Evidence suggests that CRP levels tend to decrease following periodontal treatment [11]. Other biomarkers can be titrated in the saliva and gingival crevicular fluid, including macrophage inflammatory protein-1 alpha (MIP-1*α*), interleukin-1 beta (IL−1*β*), interleukin-6 (IL-6), and matrix metalloproteinase-8 (MMP-8) [48].

A growing interest in the role of neopterin and its relationship with periodontitis has been reported in the literature [49]. Neopterin is a pteridine deriving from guanosine triphosphate involved in cell-mediated immunity, and it is considered a biomarker for macrophage activation with a regulating effect on the bioavailability of nitric oxide [50]. Its effects include the enhancement of the cytotoxic potential of activated macrophages and the activation of reactive oxygen species [51]. A role in tumor development and growth has also been hypothesized due to the role of neopterin in inducing c-fos protooncogene [52].

Neopterin can be sampled in several bodily fluids with high-performance liquid chromatography or enzyme-linked immunosorbent assay methods [53]. In GCF sampling, neopterin titration may indicate the presence of inflammation along with the detection of T-cells and other inflammatory infiltrates [51]. In urinary samples, neopterin is titrated in a ratio with creatinine. Importantly, a variability in neopterin values has been found, showing higher values in women and older individuals. Moreover, a circadian oscillation has been observed with a peak between 7.00 and 12.00 am [54]. The role of gender as a predictor of mean urinary concentration of neopterin was also confirmed by the present analysis. In our meta-analysis, only studies reporting on urinary concentration of neopterin were evaluated. However, the limited literature available hinders the validation of a direct relationship between neopterin levels and periodontitis. While it could be possible that neopterin levels may be involved in periodontitis, presumably due to macrophage activation associated both with periodontitis and neopterin levels increase, it should not be forgotten that factors other than periodontitis may contribute to neopterin fluctuations, being a very sensitive and nonspecific biomarker of cellular immune system activation [55]. Moreover, literature is extremely controversial on the effects of periodontal treatment on urinary neopterin levels, and the increase in neopterin levels has been described following periodontal therapy [33] as well as the decrease in the neopterin/creatinine ratio [29, 34]. The evidence available highlights the unmet need for clinical trials investigating the actual relationship between neopterin levels and periodontitis.

Similarly to neopterin, 8-OHdG is a biomarker for oxidative stress and carcinogenesis, which has been employed for the risk assessment of cancer and degenerative diseases [56]. 8-OHdG role as a biomarker associated with periodontitis has been previously evaluated through salivary titration, showing positive correlation with the presence of chronic periodontitis and microbial parameters [57]. Moreover, increased levels of 8-OHdG have been reported in gingival crevicular fluid of periodontally affected sites [58]. However, the study included in the present review did not highlight a correlation between urine concentrations of 8-OHdG and periodontal disease activity.

Overall, the literature appears controversial in attributing a role to both neopterin and 8-OHdG as periodontal biomarkers, highlighting the need for further clinical studies on this topic. While some studies report variations in these biomarkers' levels in periodontally affected patients versus either controls or periodontally treated patients, the level of evidence appears still limited to draw firm conclusions [59].

The present review has some limitations. First, the paucity of literature on urinary biomarkers in periodontitis allowed to evaluate only neopterin and 8-OHdG. Second, only one study reported on 8-OHdG, providing insufficient evidence on this biomarker. Moreover, the quality of the studies included was deemed at moderate/high risk of bias, which can negatively affect the quality of the results. The studies reporting on neopterin levels in periodontal patients pre- and post-treatment evaluated patients after different follow-up periods. Indeed, the comparison between periodontally affected patients versus healthy or treated groups in terms of urinary metabolite concentration as an outcome may limit the assessment of the diagnostic accuracy of the investigated biomarkers. Moreover, it was not possible to assess the potential role of confounders contributing to systemic inflammation. The included studies enrolled relatively limited samples, thus highlighting the need for further evaluation on larger cohorts.

Nevertheless, a trend toward an increase in urinary neopterin can be hypothesized in the presence of untreated periodontitis, as the literature available suggests a normalization of neopterin levels following periodontal treatment. Conversely, the evidence on 8-OHdG is still extremely scarce and cannot be validated.

## 5. Conclusions

The evidence available from the present review aims at raising awareness on the potential role of urinary biomarkers for the diagnosis, prognosis, and monitoring of periodontitis, as to date there is insufficient evidence to draw firm conclusions. Further studies assessing urinary biomarker variations in the course of periodontitis compared to treated or healthy subjects are advised to enrich the body of literature on this topic.

## Figures and Tables

**Table 1:** PRISMA checklist for systematic review reporting.

Section/topic	#	Checklist item	Adherence	Pages
Title	1	Identify the report as a systematic review, meta-analysis, or both	Yes	1
Abstract				
Structured summary	2	Provide a structured summary including, as applicable: background; objectives; data sources; study eligibility criteria, participants, and interventions; study appraisal and synthesis methods; results; limitations; conclusions and implications of key findings; systematic review registration number	Yes	1
Introduction				
Rationale	3	Describe the rationale for the review in the context of what is already known	Yes	3
Objectives	4	Provide an explicit statement of questions being addressed with reference to participants, interventions, comparisons, outcomes, and study design (PICOS)	Yes	4
Methods				
Protocol and registration	5	Indicate if a review protocol exists, if and where it can be accessed (e.g., web address), and, if available, provide registration information including registration number	Yes	4
Eligibility criteria	6	Specify study characteristics (e.g., PICOS, length of follow-up) and report characteristics (e.g., years considered, language, publication status) used as criteria for eligibility, giving rationale	Yes	4–5
Information sources	7	Describe all information sources (e.g., databases with dates of coverage, contact with study authors to identify additional studies) in the search and date last searched	Yes	5
Search	8	Present full electronic search strategy for at least one database, including any limits used, such that it could be repeated	Yes	6–7
Study selection	9	State the process for selecting studies (i.e., screening, eligibility, included in systematic review, and, if applicable, included in the meta-analysis)	Yes	7
Data collection process	10	Describe the method of data extraction from reports (e.g., piloted forms, independently, in duplicate) and any processes for obtaining and confirming data from investigators	Yes	7
Data items	11	List and define all variables for which data were sought (e.g., PICOS, funding sources) and any assumptions and simplifications made	Yes	7–8
Risk of bias in individual studies	12	Describe methods used for assessing risk of bias of individual studies (including specification of whether this was done at the study or outcome level), and how this information is to be used in any data synthesis	Yes	8
Summary measures	13	State the principal summary measures (e.g., risk ratio, difference in means); describe the methods of handling data and combining results of studies, if done, including measures of consistency (e.g., *I*^2^) for each meta-analysis	Yes	7–9
Synthesis of results	14	—	Yes	9
Risk of bias across studies	15	Specify any assessment of risk of bias that may affect the cumulative evidence (e.g., publication bias, selective reporting within studies)	Yes	9
Additional analyses	16	Describe methods of additional analyses (e.g., sensitivity or subgroup analyses, meta-regression), if done, indicating which were prespecified	Yes	9
Results				
Study selection	17	Give numbers of studies screened, assessed for eligibility, and included in the review, with reasons for exclusions at each stage, ideally with a flow diagram	Yes	Table 2
Study characteristics	18	For each study, present characteristics for which data were extracted (e.g., study size, PICOS, follow-up period) and provide the citations	Yes	Table 1
Risk of bias within studies	19	Present data on risk of bias of each study and, if available, any outcome level assessment (see Item 12)	Yes	Figure 1
Results of individual studies	20	For all outcomes considered (benefits or harms), present, for each study: (a) simple summary data for each intervention group and (b) effect estimates and confidence intervals, ideally with a forest plot	Yes	Figures 2, 3, 4, and 5
Synthesis of results	21	Present results of each meta-analysis done, including confidence intervals and measures of consistency	Yes	10–11
Risk of bias across studies	22	Present results of any assessment of risk of bias across studies (see Item 15)	Yes	10–11
Additional analysis	23	Give results of additional analyses, if done (e.g., sensitivity or subgroup analyses, meta-regression (see Item 16))	Yes	10–11
Discussion				
Summary of evidence	24	Summarize the main findings including the strength of evidence for each main outcome; consider their relevance to key groups (e.g., healthcare providers, users, and policymakers)	Yes	11
Limitations	25	Discuss limitations at study and outcome level (e.g., risk of bias), and at review level (e.g., incomplete retrieval of identified research, reporting bias)	Yes	13
Conclusions	26	Provide a general interpretation of the results in the context of other evidence, and implications for future research	Yes	13
Funding				
Funding	27	Describe sources of funding for the systematic review and other support (e.g., supply of data); role of funders for the systematic review	Yes	13

**Table 2:** Reasons for study exclusion following full-text analysis.

Nonperiodontal patients	Absence of biomarkers	Other diseases/not periodontal biomarkers	Same data from other study
Cooke et al. [13] Ojeda et al. [14]	Matsumoto et al. [15] Yamori et al. [16] Yoshihara et al. [17] Yoshihara et al. [18]	Brotto et. al. [19] Grubbs et al. [20] Ioannidou et al. [21] Kang et al. [22] Liu K et al. [23] Mesa et al. [24] Nakajima et al. [12] Schulze-Späte et al. [25] Vachhani et al. [26] von Wowern et al. [27] Wangerin et al. [28]	Prasanna et al. [29]

**Table 3:** Electronic search strategy.

Electronic search strategy
PubMed: (“periodontal disease”(Mesh) OR “periodontitis”(Mesh) OR “gingivitis”(Mesh) OR “periodontal disease” OR “periodontitis” OR “gingivitis”) AND (“urine” OR “urinary” OR “urinalysis”) Embase: (“periodontal disease”/exp OR “periodontitis”/exp OR gingivitis ^*∗*^) AND (“urine”/exp OR “urinary”/exp OR urinalysis ^*∗*^) Cochrane: #1 MeSH descriptor: (Periodontal diseases) explode all trees #2 MeSH descriptor: (Periodontitis) explode all trees #3 MeSH descriptor: (Gingivitis) explode all trees #4 #1 OR #2 OR #3 #5 #4 AND “urine” #6 #4 AND “urinary” #7 #4 AND “urinalysis”

**Table 4:** Synthesis of the included studies.

Authors	Year	Study design	Follow-up (months)	Metabolites	Unite of measure	Sample size	Female (%)	Concentrations							
								Case group				Control			
								Baseline		Follow-up time		Baseline		Follow-up time	
								Mean	SD	Mean	SD	Mean	SD	Mean	SD
Andreu et al. [30]	2021	Cross-sectional	—	8-OHdG	Creatinine (*µ*g/g)	70	61.4	10.9 Stage II 9.17 Stage III 9.84 Stage IV	3.05 2.05 3.21	—	—	—	—	—	—
Bodur et al. [31]	2003	Observational study case control	6	Neopterin	Neopterin (*μ* mol)/creatinine (mol)	16	68.8	168.1	20.21	310.1	39.82	188.5	30.98	—	—
Heneberk et al. [32]	2022	Observational study case control	3	Neopterin	Neopterin (*μ* mol)/creatinine (mol)	25	—	210.96 (183.60–282.91)	—	237.87 (202.46–266.80)	—	180.59 (133.97–220.22)	—	—	—
Ozmeriç et al. [33]	2002	Observational study case control	—	Neopterin	Neopterin (*μ* mol)/creatinine (mol)	29	55.1	235.77	405.31	—	—	225.45	100.72	—	—
Prasanna and Sumadhura [34]	2019	Cross-sectional interventional study	3	Neopterin	Neopterin (*μ* mol)/creatinine (mol)	30	100.0	348.06	61.45	278.01	53.53	—	—	—	—
Vrecko et al. [35]	1997	Observational cohort study	—	Neopterin	Neopterin (*μ* mol)/creatinine (mol)	29	37.9	102	48	—	—	—	—	—	—

## Data Availability

The data presented in this study are openly available.
